# Assessment of safety and efficacy of risdiplam treatment in adults with spinal muscular atrophy

**DOI:** 10.3389/fneur.2025.1694037

**Published:** 2026-01-14

**Authors:** Andrea Jaworek, Kathryn Jira, Matti Allen, Songzhu Zhao, Kristina Kelly, Trevor Moravec, Marco Tellez, Sarah Heintzman, Jerold Reynolds, Gary Sterling, Stephen J. Kolb, William David Arnold, Bakri Elsheikh

**Affiliations:** 1 Division of Neuromuscular Disorders, Department of Neurology, College of Medicine, The Ohio State University Wexner Medical Center, Columbus, OH, United States; 2 Ottawa Hospital Research Institute, Ottawa, ON, Canada; 3 Department of Biomedical Informatics and Center for Biostatistics, The Ohio State University, Columbus, OH, United States; 4 NextGen Precision Health, University of Missouri, Columbia, MO, United States; 5 Department of Neurology, Knox Community Hospital, Mount Vernon, OH, United States

**Keywords:** risdiplam, SMA, adults, outcomes, safety, effect

## Abstract

**Introduction:**

Risdiplam has been shown to be safe, well tolerated, and improves or stabilizes motor function in individuals with SMA, but limited published data exists for adults. The aim of this study was to assess the efficacy, safety, and tolerability of risdiplam treatment for adults with SMA.

**Methods:**

We conducted a retrospective chart review on adult patients with 5q-SMA who received risdiplam for a minimum of 6 months, including both treatment naïve and those who switched from nusinersen. Baseline demographic data was collected and outcomes included the Revised Upper Limb Module, Children’s Hospital of Philadelphia Adult Test of Neuromuscular Disorders (CHOP-ATEND), six-minute walk test, Hammersmith Functional Motor Scale-Expanded, and forced vital capacity. Assessments were performed at baseline, 6, 12, and 24 months. Self-reported adverse effects were recorded. Linear mixed models were used for analysis.

**Results:**

Eighteen patients (mean age 41.11 years) met inclusion criteria. CHOP-ATEND scores increased at 12 (+1.99, *p* = 0.030) and 24 months (+2.12; *p* = 0.042), while all other outcomes showed stability. The most common self-reported adverse effects were gastrointestinal issues. Serious adverse events included pneumonia, appendicitis, and femur and tibia/fibula fractures. The latter two were considered unlikely related to treatment.

**Discussion:**

Risdiplam is overall safe and well-tolerated up to 24 months in adults with SMA. The treatment resulted in improvement or stabilization of motor and respiratory function in non-ambulatory and ambulatory patients. Improvement on the CHOP-ATEND suggests it may be a sensitive marker of change. Longer-term follow-up is needed to understand the impact of risdiplam in adults with SMA.

## 1 Introduction

Spinal muscular atrophy (SMA) is a genetic disorder characterized by the degeneration of anterior horn cells leading to muscle atrophy and weakness (1). The incidence of SMA is 1:15,000 live births (2). The severity of SMA varies widely, ranging from early infant death to normal adult life with mild weakness (3). Common symptoms include hypotonia, muscular atrophy, and proximal muscle weakness, with more severe lower limb involvement (4). In the most severe phenotypes, bulbar weakness, respiratory difficulties, and scoliosis are also observed. Therefore, patients with SMA often require comprehensive medical care involving a multidisciplinary approach (3).

SMA is an autosomal-recessive disease caused by a deficiency in the survival motor neuron (SMN) protein due to mutation in the *SMN1* gene (5). Individuals with SMA rely on a backup *SMN2* gene to produce a small amount of SMN protein necessary for survival (1). Disease severity is modified by the number of the *SMN2* gene copies (5). Those with more severe phenotypes tend to have fewer copies of *SMN2* (6).

There are three FDA-approved medications to modify the disease course of SMA: nusinersen, onasemnogene abeparvovec, and risdiplam (7). Among these, only oral risdiplam and intrathecal nusinersen are approved for the adult population. There is real world data supporting the safety and effect of nusinersen across a wide age range including adults (8–15). Similar data are emerging for risdiplam, however, these are more heavily focused on infants and children with relatively limited long-term real-world evidence in adults (16–27).

Risdiplam is a small molecule that is orally administered and modifies *SMN2* pre-messenger RNA splicing, increasing the levels of full-length, functional SMN protein (19). Its appeal in adults with SMA lies in being an oral medication, unlike nusinersen, which requires intrathecal injection (a procedure that can be challenging for patients with significant spinal deformity or a history of spinal surgeries). Additionally, risdiplam does not require routine blood or urine laboratory testing. Common side effects in adults include infection, rash, and diarrhea. Other safety information includes precautions regarding pregnancy, male fertility, and potential interactions with other medications. Adult research is fairly limited in duration of the trial, number of subjects, and variability in ability levels. Given the paucity of the data on risdiplam in adults with SMA, further research is needed in individuals 18 years or older. This study aimed to assess the safety and efficacy of risdiplam in a cohort of adults with SMA.

## 2 Materials and methods

### 2.1 Study design

Data was collected retrospectively through an IRB approved study conducted at The Ohio State University Wexner Medical Center (OSU). Medical records of adult patients with SMA receiving care for SMA management at OSU between 2017 and 2024 were reviewed using the Integrated Healthcare Information System (IHIS).

### 2.2 Study population

Individuals (≥18 years) with genetically confirmed 5q-SMA who had been on risdiplam for at least 6 months were included in the study. The study included both treatment naïve patients and those transitioning from nusinersen. A total of 53 medical charts were initially reviewed; however, 29 patients were excluded due to ongoing treatment with nusinersen, two were lost to follow-up, one was not on a disease modifying treatment, one due to comorbidities of a central nervous system (CNS) glioma, another with insufficient data due to being a transfer from another health care system, and one had just started risdiplam with not enough data time points yet. This resulted in 18 patients meeting the inclusion criteria.

### 2.3 Study overview

Assessment data were extracted and reviewed at baseline, 6 months, 12 months, and 24 months. Retrospective data included age, sex, age at symptom onset, *SMN2* copy number, and body mass index. Clinical outcomes collected included the Revised Upper Limb Module (RULM), Hammersmith Functional Motor Scale-Expanded (HFMSE), and the Children’s Hospital of Philadelphia Adult Test of Neuromuscular Disorders (CHOP-ATEND) for non-ambulatory patients, and the six-minute walk test (6MWT), RULM, and HFMSE for ambulatory patients. All functional outcome measures were administered by a physical therapist with neuromuscular expertise. Forced Vital Capacity (FVC) and self-reported adverse effects were also collected. For this study, an ambulatory individual was defined as someone who could ambulate at least 10 meters with or without an assistive device. This was determined by their documented status in the medical chart prior to starting risdiplam.

The RULM has been shown to have good reliability and validity in both non-ambulatory and ambulatory individuals with SMA (28). The CHOP-ATEND, a revised scale from the original Children’s Hospital of Philadelphia Infant Test of Neuromuscular Disorders (CHOP-INTEND), was also used in this study. This revised version for adults excludes items that cannot be performed with the older population (items 11, 15, and 16) and accounts for the severity of contractures that is often seen in adults (11). The 6MWT, a widely used outcome measure for individuals with neuromuscular conditions, has also proven to be a valid and reliable outcome measure for pediatric and adult patients with SMA (29). The HFMSE has been proven to be a valid and time-efficient outcome measure in non-ambulatory and ambulatory adults with SMA (30).

### 2.4 Statistical analysis

Patients’ demographic and clinical characteristics were summarized using means (standard deviations) for continuous variables and frequencies (proportions) for categorical variables. Linear mixed models with random intercept for each participant and unstructured covariance matrix were employed to examine the changes of outcome measure over time. Differences between each time point from baseline with 95% confidence intervals (CI) were reported. Spaghetti plots were employed to depict individual changes across time. Self-reported adverse effects were investigated using a descriptive analysis. All statistical analyses were conducted using SAS 9.4, and a *p*-value of less than 0.05 was considered statistically significant.

## 3 Results

### 3.1 Demographics and baseline assessments

A total of 18 patients met the inclusion criteria and were analyzed through 24 months post-risdiplam treatment. Table 1 summarizes participant demographics including age, sex, SMA genotype and phenotype characteristics, along with baseline functional assessments. The cohort mean age was 41.11 ± 14.83 years, with 61% women. Most patients (83%) were non-ambulatory, and 78% had 3 *SMN2* copies. The majority of our patients had scoliosis (83%) and 60% of those patients had spinal surgery. Two patients were ventilator dependent while one of these patients also had a tracheostomy. Four patients were receiving non-invasive ventilation support with positive airway pressure machines.

**Table 1:** Baseline characteristics.

Variable	Level	Total (*n* = 18)
Age	Mean (SD)	Missing = 0
	(min, max)	41.11 (14.83)
		(23, 71)
Sex	F	11 (61.11%)
	M	7 (38.89%)
BMI	Mean (SD)	Missing = 9
	(min, max)	28.61 (8.95)
		(18.66, 43.6)
Disease duration	Median [IQR]	Missing = 2
	(min, max)	31 [24.45, 48.45]
		(20, 69)
*SMN2* copies	Missing	2 (11.11%)
	3	14 (77.78%)
	4	2 (11.11%)
Previous nusinersen	No	7 (38.89%)
	Yes	11 (61.11%)
Ambulatory status	Ambulatory	3 (16.67%)
	Non-ambulatory	15 (83.33%)
HFMSE	Median [IQR]	Missing = 11
	(min, max)	19 [6, 56]
		(2, 57)
RULM	Median [IQR]	Missing = 4
	(min, max)	20 [7, 29]
		(2, 37)
CHOP	Median [IQR]	Missing = 13
	(min, max)	16 [14, 25]
		(1, 29)
6MWT	Median [IQR]	Missing = 14
	(min, max)	239.65 [42.1, 437.5]
		(4.9, 475)
FVC	Median [IQR]	Missing = 8
	(min, max)	1.69 [1.31, 2.56]
		(0.66, 5.23)
FVC predicted	Median [IQR]	Missing = 8
	(min, max)	0.44 [0.4, 0.74]
		(0.21, 1.03)

Baseline characteristics for all participants.

The baseline HFMSE was scorable in only about one third of our non-ambulatory patients, underscoring the severity of the phenotype of our patient population. The median RULM score was 20 (IQR 7–29) for all participants. The CHOP-ATEND scores ranged from 1–29 among five patients who had the baseline assessment. The baseline 6MWT had a large variation of 79.3 to 475 meters for all participants who met our ambulatory definition. FVC for all participants was a median of 1.69 (IQR 1.31–2.56).

### 3.2 Longitudinal outcome assessments

**Figure 1:**
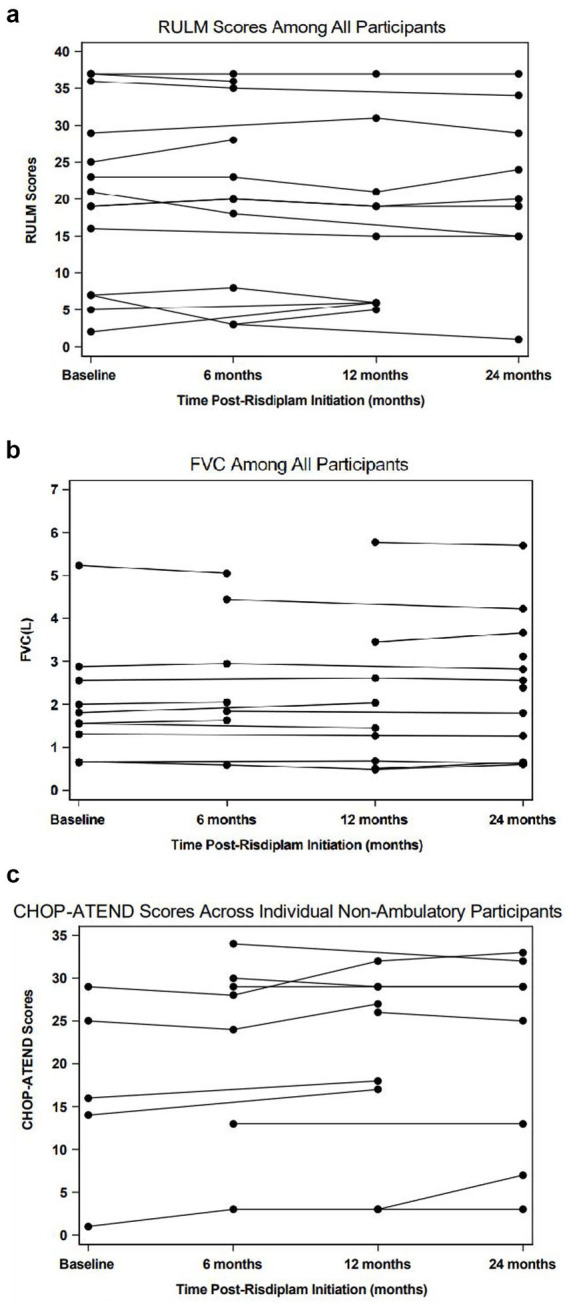
Spaghetti plots depicting longitudinal change of various motor and respiratory outcomes measures. **(1A)**: RULM; **(1B)**: FVC; **(1C)**: CHOP-ATEND.

Functional outcome measures and pulmonary function results for all participants at baseline, 6, 12, and 24 months are summarized in Table 2. The HFMSE and RULM for all participants remained stable over the 24-month period, indicating overall functional stability. RULM results are depicted in Figure 1a. Similarly, FVC remained stable, reflecting sustained ventilatory muscle function throughout the study duration (Figure 1b).

**Table 2:** Longitudinal outcome measure results over 24-month duration for all participants.

Measure	Time/Comparison	Number (*N* = 18)	Estimate (baseline/change from baseline)	95% CI	*p*-value
Functional scales					
HFMSE	Baseline	7	31.97	(24.63, 39.31)	
	6 months	6	0.73	(−1.30, 2.76)	0.429
	12 months	3	2.47	(−0.28, 5.21)	0.072
	24 months	2	1.4	(−1.87, 4.67)	0.353
RULM	Baseline	14	19.17	(13.44, 24.90)	
	6 months	11	0.1	(−1.13, 1.33)	0.869
	12 months	10	−0.2	(−1.44, 1.05)	0.748
	24 months	9	−0.73	(−2.06, 0.59)	0.266
CHOP-ATEND	Baseline	5	19.05	(12.90, 25.21)	
	6 months	7	1.32	(−0.69, 3.33)	0.182
	12 months	9	1.99	(0.22, 3.76)	0.03
	24 months	8	2.12	(0.08, 4.15)	0.042
6MWT	Baseline	3	290.68	(−1,620, 2201.0)	
	6 months	2	12.42	(−70.16, 95.00)	0.665
	12 months	2	15.3	(−68.30, 98.89)	0.601
	24 months	2	9.5	(−74.10, 93.09)	0.742
Pulmonary function					
FVC	Baseline	10	2.41	(1.83, 2.99)	
	6 months	7	0.03	(−0.06, 0.13)	0.488
	12 months	9	−0.04	(−0.13, 0.05)	0.394
	24 months	12	−0.03	(−0.12, 0.06)	0.557
FVC predicted	Baseline	10	0.61	(0.48, 0.74)	
	6 months	7	0.02	(−0.01, 0.05)	0.164
	12 months	9	0	(−0.03, 0.02)	0.798
	24 months	12	0	(−0.03, 0.03)	0.928

The analysis of the 6MWT was conducted among ambulatory patients and adjusted for prior use of nusinersen. The CHOP-ATEND analysis was limited to non-ambulatory patients and also adjusted for prior nusinersen use. For all other outcomes, analyses included all patients and models were adjusted for both prior nusinersen use and ambulatory status.

CHOP-ATEND scores showed a statistically significant increase at 12 (+1.99, *p* = 0.030) and 24 months (+2.12, *p* = 0.042) (Figure 1c). Of the five patients that completed the baseline visit, 100% of these patients increased their score from the baseline visit to their final visit by 2 to 4 points.

Two of the three ambulatory participants showed increases of 41.2 and 27.6 meters from their baseline to final visit for the 6MWT, both exceeding the minimal detectable change (MDC) score of >24 meters established for the SMA population (29). The third patient, who was more functionally impaired at baseline, demonstrated a decrease of 29.8 meters. This individual had the shortest baseline distance of 79.3 meters, walking >300 meters less than the other two participants.

### 3.3 Tolerability and adverse events

Based on the records review, it appears that risdiplam was overall well tolerated with only one patient discontinuing the medication after approximately 24 months of use due to side effects. They reported increased muscle pain, weakness, and mood changes. Five patients experienced serious adverse events, and 11 patients reported adverse events. Serious adverse events included hospitalizations related to pneumonia in one patient, fractures in two patients, appendicitis in one patient, and multiple hospitalizations in another patient related to pain. The two events related to fractures included one who had a tibia-fibula fracture following a motor vehicle accident and the other suffered a femur fracture from falling out of a wheelchair during a transfer. Given the documented circumstances, these were deemed unrelated to risdiplam. Similarly, the patient hospitalized for pneumonia (with confirmed COVID-19) and the patient with appendicitis experienced events consistent with acute illnesses that can occur in the general population. Given the nature of these events and their occurrence more than a year after starting treatment, they were considered unlikely related to risdiplam. Another patient was hospitalized numerous times for various circumstances from pain. Overall, there were eight hospitalizations among five of the 18 patients.

The most common documented self-reported adverse effects included gastrointestinal issues, observed in seven patients (39%). Specifically, diarrhea was reported in six out of the 18 patients (33%), and nausea in one patient (6%). Two of the patients with diarrhea also reported episodes of constipation. Other self-reported symptoms included headache in 11% of patients. Musculoskeletal pain was reported in six out of the 18 patients (33%), collected by chart review; however, it should be noted that pain is multifactorial in patients with SMA and could be due to issues such as positioning, contractures, or fatigue. Of note, three patients (50%) did not report pain at the baseline visit. Eight patients (44%) reported no side effects.

## 4 Discussion

### 4.1 Outcome measures

Our study contributes to the evolving literature on the safety and efficacy of risdiplam in adult patients with SMA with a wide range of functional abilities. Despite this variability, our patients predominantly represent a severe phenotype, as evident by the majority of participants unable to perform the HFMSE.

This severity is further underscored by the significant changes observed in the CHOP-ATEND scores, a scale specifically designed for assessing the most severely affected patients with SMA. One study found that scores increased on the CHOP-INTEND after 10 months duration on risdiplam (22) and our study confirmed this for a longer duration at 12 and 24 months. Our results revealing stability in the HFMSE and RULM align with another study that showed no improvement for the HFMSE but it did observe improvement in the RULM for non-ambulatory adults over 12 months (24). The stability observed in HFMSE and RULM scores in our study contrasts sharply with the expected natural history of SMA. Typically, non-ambulatory and ambulatory individuals (including adults) with SMA experience an annual decline of 0.5 to 1 point on the HFMSE and 0.79 points on the RULM (31, 32). This data differs with our noted stabilization for the HFMSE and the RULM. Additionally, FVC is expected to decline by 0.2 to 1.4% annually in individuals with SMA, also including adults (33), and our FVC results demonstrated stability at 24 months. A study on non-ambulant and ambulatory adult individuals with SMA observed meaningful improvements in 22.6% of patients and stabilization of motor functions in 61% (25) and our study contributes to this real-world data. Another study investigated 14 individuals with SMA and found improvements in various functional outcome measures, however the median age was 11 years younger than our patient population (26). Similar to our study, the ambulatory participants comprised a smaller population. The Jewelfish study is one of the largest trials investigating the effects of risdiplam in pediatric and adult individuals (27). However, it cannot be accurately compared to ours because it was not stratified by age. It must be emphasized that Sunfish found that younger individuals had a greater improvement in outcome measures while stabilization was mostly seen for older individuals (16, 17). This supports our findings that most outcome measures stabilized for the adult population, apart from the CHOP-ATEND for our study. This highlights that it is important to track change in severe phenotypes in adults.

While our findings argue for the utility of the CHOP-ATEND as an appropriate outcome measure for this population, particularly in those with the most severe manifestations of the disease, there is a newer outcome measure (based on the CHOP-ATEND), called the Adapted Test of Neuromuscular Disorders (ATEND). It is a functional motor outcome assessment for individuals with a neuromuscular disorder who are unable to sit or transfer out of the wheelchair. The ATEND is undergoing a validity and reliability study and its distinguishing factor of being able to assess a patient in their wheelchair decreases burden on the patient and offers promise to clinicians.

Overall, the lack of expected decline in motor function and respiratory capacity in individuals in our study suggest that risdiplam plays a notable role in stabilizing the disease in adults with severe SMA phenotypes.

### 4.2 Previous treatment

There is currently no direct comparison between nusinersen and risdiplam in adults with SMA. As a result, patients, specifically adults, typically base their decision on which medication to use on factors such as the route of administration, side effect profile, and their personal experience with a specific treatment. In the authors’ practice, ambulatory subjects represent around a third of all patients. However, in this study, ambulatory subjects comprised only about 17% of the population. The percentage of the adult ambulatory population varies across studies. This has shown to range from approximately 37–42% (8, 11, 34) in nusinersen studies and less than 10% in the risdiplam studies (23, 26). The lower number of ambulatory subjects in the risdiplam studies could be partially attributed to the challenges of intrathecal access in non-ambulatory patients, making an oral route like risdiplam more appealing. Furthermore, ambulatory patients who started nusinersen treatment prior to risdiplam approval may have opted not to switch medications if they noted improvement with their current treatment, which limits the overall number of ambulatory patients on risdiplam. In our study, a total of 11 patients were previously on nusinersen (two ambulatory and nine non-ambulatory).

The main reason for the switch was related to the burden of the route of administration with nusinersen and post lumbar puncture headache and back pain. It would be of benefit to investigate a larger cohort of ambulatory participants in the future to better characterize the effects of risdiplam in individuals with milder forms of SMA.

### 4.3 Side effects

The side effects reported in this study were consistent with those documented in previous reports, including pneumonia, diarrhea, headache, and pain. It is worth noting that although pain and headache reported by patients in this cohort are not listed in the drug insert^1^ (35) they have been observed in real-world data. Additionally, while the two serious adverse events related to fractures were provoked and likely unrelated to the drug, these findings underscore the importance of monitoring and maintaining bone health in this population, as spontaneous fractures are a known concern in individuals with SMA (36–38).

### 4.4 Limitations

This study has several important limitations. The retrospective nature of the study, combined with a small sample size, particularly within the ambulatory cohort, limits the generalizability of the findings. Additionally, the study was conducted during the COVID-19 pandemic, which resulted in missed assessments and disruptions in data collection. Many patients were unable to attend in-person visits, leading to gaps in the outcome measure assessments and limiting the overall data set.

Given the small number of participants and missing data at specific time points, our analyses are primarily descriptive and exploratory, focusing on observed trends rather than drawing definitive conclusions. Preexisting conditions prior to treatment were not captured in this study, which prevents direct comparisons of the participants’ health status before and after treatment and limits the ability to assess causal relationships between adverse events and the treatment.

Despite these limitations, our findings support a favorable safety and efficacy profile of risdiplam across a broad age range and phenotypic severity in adults with SMA. The study also highlights the potential critical role of sensitive outcome measures, such as the CHOP-ATEND, particularly for assessing severe phenotypes in non-ambulatory patients. However, this should be further assessed due to our small population. Research is currently underway to optimize similar outcome measures, which are essential for accurately tracking disease progression and treatment effects. The continued collection of real-world data is essential for gaining a deeper understanding of the long-term impact of risdiplam, particularly beyond the two-year mark, on adults with SMA.

## Data Availability

The raw data supporting the conclusions of this article will be made available by the authors, without undue reservation.

## Footnotes

1
https://www.accessdata.fda.gov/drugsatfda_docs/label/2024/213535s013lbl.pdf
